# Molecular mechanisms underlying *Fagopyrum dibotrys*-derived nanovesicles induced ferroptosis in hepatocellular carcinoma: a dual-pathway analysis of lipid peroxidation and mitochondrial damage

**DOI:** 10.3389/fphar.2025.1636149

**Published:** 2025-06-26

**Authors:** Ling Wu, Hongyao Chen, Jingting Zhang, Jincheng Tang, Zhibin Wang, Peisen Xue, Wenhui Gao, Renyi Yang, Puhua Zeng

**Affiliations:** ^1^ Hunan Provincial Hospital of Integrated Traditional Chinese and Western Medicine, Hunan Academy of Chinese Medicine, Changsha, Hunan, China; ^2^ Hunan Provincial Key Laboratory of Traditional Chinese Medicine Formula and Syndrome Research in Translational Medicine, Hunan University of Chinese Medicine, Changsha, Hunan, China; ^3^ Cancer Research Institute of Hunan Academy of Traditional Chinese Medicine, Changsha, Hunan, China

**Keywords:** *Fagopyrum dibotrys*, nanovesicles, hepatocellular carcinoma, ferroptosis, *in vitro* and *in vivo* experiments

## Abstract

**Background:**

Hepatocellular carcinoma (HCC) is a prevalent malignant tumor globally, with high incidence and mortality rates that seriously endanger human health. While traditional therapeutic approaches have demonstrated some efficacy in controlling disease progression, they are still fraught with numerous limitations. In recent years, plant-derived nanovesicles have garnered significant attention owing to their distinctive biological activities and promising antitumor characteristics. The effects of *Fagopyrum dibotrys*, a plant with various medicinal values, and its-derived nanovesicles (FdNVs) on HCC cells have not been clarified.

**Objective:**

This study aimed to explore the inhibitory effects of FdNVs on human HCC cells and subcutaneous xenograft tumors, as well as the underlying molecular mechanisms.

**Methods:**

FdNVs were isolated and purified through ultracentrifugation, characterized via Nanoparticle Tracking Analysis (NTA) and Transmission Electron Microscopy (TEM), and subsequently evaluated *in vitro* using the HepG2 HCC cell line to assess their effects on proliferation (via cell viability, EdU, and colony formation assays), migration (wound healing assay), and invasion (Transwell assay), while mitochondrial ultrastructural changes were examined by TEM, intracellular ROS and Fe^2+^ levels were measured fluorometrically, oxidative stress markers (GSH and MDA) were quantified colorimetrically, ferroptosis-related mRNA and protein expression were analyzed by RT-qPCR and Western blot, followed by *in vivo* validation of their antitumor efficacy in a nude mouse HepG2 xenograft model.

**Results:**

*In vitro* studies demonstrated that FdNVs dose-dependently suppressed HepG2 cell proliferation, motility, and invasive capacity. Mechanistic investigations revealed that this inhibitory effect was mediated through ferroptosis activation, supported by the following observations: elevated intracellular ferrous iron (Fe^2+^) and reactive oxygen species (ROS), reduced glutathione (GSH) content, disrupted mitochondrial ultrastructure, and modulated expression of key ferroptosis regulators—including upregulation of pro-ferroptotic proteins (p53 and ALOX15) and downregulation of anti-ferroptotic factors (xCT and GPX4). Furthermore, *in vivo* studies validated the tumor-suppressive role of FdNVs, confirming their capacity to trigger ferroptosis in HepG2 xenografts.

**Conclusion:**

FdNVs inhibited the proliferation, migration and invasion of HCC cells by inducing iron death, and their anti-tumor mechanism involved the regulation of iron death-related genes and proteins.

## Introduction

Hepatocellular carcinoma (HCC) represents a major global health challenge, ranking as the sixth most commonly diagnosed malignancy and the third leading cause of cancer mortality worldwide according to GLOBOCAN 2022 estimates, with an annual incidence of 865,000 cases (4.3% of all cancers) and 758,000 deaths (7.8% of cancer fatalities) (Bray et al., 2024). The disease disproportionately affects developing regions and arises from diverse etiological factors, including viral hepatitis infections, alcohol-related liver damage, and metabolic dysfunction-associated steatotic liver disease (Wang et al., 2020). The prognosis for advanced-stage HCC remains poor, largely due to the lack of reliable early detection methods. Existing treatments for late-stage disease—including surgical intervention, systemic chemotherapy, radiation therapy, and molecular-targeted agents—are frequently constrained by suboptimal response rates and substantial adverse effects, highlighting a critical need for more effective therapeutic approaches (Akce et al., 2022). In this context, traditional Chinese medicine (TCM) has emerged as a promising complementary strategy, offering distinct advantages through its multi-targeted regulatory mechanisms and favorable safety characteristics. These properties underscore the importance of investigating mechanism-based innovative therapies to overcome current clinical limitations in HCC management.

In recent years, nanotechnology has made significant strides in the field of cancer therapy. Nanomaterials have demonstrated the ability to enhance drug targeting and biocompatibility, as well as regulate drug release mechanisms, thereby improving therapeutic efficacy and reducing side effects (Zhu et al., 2024; Yen et al., 2025; Zou et al., 2025). Exosome-like nanoparticles derived from botanical sources (PDENs) have emerged as promising therapeutic candidates, distinguished by their wide availability, excellent biocompatibility, potent biological activity, and minimal immune reactivity, characteristics that establish their potential as innovative mediators of cellular crosstalk (Ou et al., 2023). Research indicates that PDENs can modulate tumor growth and metastasis by releasing bioactive molecules, offering advantages in immunomodulation, drug delivery, and targeting for the treatment of malignant tumors (Dad et al., 2021). *Fagopyrum dibotrys*, a perennial herb widely used in traditional Chinese medicine, is rich in bioactive constituents, including flavonoids and polyphenols. These compounds have demonstrated significant pharmacological effects, such as anti-inflammatory, antioxidant, and antitumor activities, as supported by experimental evidence (Zhang et al., 2023). As a novel iron-mediated regulated cell death mechanism, ferroptosis has gained considerable attention in oncology research due to its tumor-selective cytotoxic effects mediated by lipid peroxide accumulation. Induction of ferroptosis can effectively inhibit tumor cell proliferation by modulating intracellular iron metabolism, lipid peroxidation, and the antioxidant response (Stockwell et al., 2017; Zhou et al., 2025). Studies have shown that *Fagopyrum dibotrys* extracts exert anti-cancer effects by suppressing inflammatory mediators and promoting apoptosis (Jing et al., 2016); however, their potential role in regulating ferroptosis remains unexplored. In our previous work, high-performance liquid chromatography–mass spectrometry (HPLC–MS) was employed by our group to systematically analyze the bioactive components of FdNVs. This analysis revealed a chemically diverse profile, including fatty acyls, glycosides, amino acids, alkaloids, terpenoids, and flavonoids—many of which have documented anti-inflammatory, antioxidant, and anticancer properties. These findings not only underscore the therapeutic potential of FdNVs but also provide mechanistic insights into their biological activity. Building on these observations, we employed FdNVs, known for their safety and biocompatibility, to investigate their potential to suppress HCC through ferroptosis-related pathways.

This study focuses on systematically investigating the ferroptosis-inducing effects of FdNVs in HCC cells and elucidating their underlying mechanisms. Utilizing complementary experimental approaches including both HCC cell culture systems and murine xenograft models, our investigation systematically characterized the ferroptosis-inducing capacity of FdNVs in HCC and elucidated the underlying molecular mechanisms. Specifically, we explored whether FdNVs promote intracellular iron accumulation, exacerbate lipid peroxidation, and suppress antioxidant enzyme system activity. Additionally, we assessed the antitumor efficacy of FdNVs in HCC-bearing mice and investigated their potential for clinical translation. This study aims to provide experimental evidence supporting the development of novel ferroptosis-based therapeutic strategies for HCC.

## Materials

### Cell lines

The human HCC cell line HepG2 (Batch No.: CL-0103) was obtained from Wuhan Pricella Biotechnology Co., Ltd. Cells were propagated in Dulbecco’s Modified Eagle Medium (DMEM) containing 10% fetal bovine serum (FBS) and 1% penicillin-streptomycin antibiotic solution. Cultures were incubated at 37°C in a 5% CO_2_ humidified atmosphere. Routine passaging was conducted at 1–2 days intervals, with experimental procedures exclusively utilizing cells in logarithmic growth phase demonstrating >95% viability as assessed by trypan blue exclusion.

### Animals

Male BALB/c nude mice (4-week-old, SPF grade) were procured from Hunan Slake Jingda Laboratory Animal Co., Ltd. (license number: SCXK 2019–0004). The study protocol received full approval from the Institutional Animal Care and Use Committee at Hunan University of Chinese Medicine, with all procedures performed in compliance with national ethical guidelines for laboratory animal welfare (Ministry of Science and Technology, China). Animals were maintained in the university’s AAALAC-accredited facility under standardized environmental conditions (22°C ± 1°C, 55% ± 5% humidity) with controlled 12-h photoperiods and *ad libitum* access to sterilized feed and water. A total of 25 mice were utilized, with 5 mice allocated to each experimental group. To ensure the reliability of the results, each experiment was repeated three times. Following a 7-day environmental adaptation phase, tumor xenografts were established via subcutaneous inoculation of 1 × 10^7^ HepG2 cells in the right flank region.

### Reagents and chemicals

Fresh *F. dibotrys* rhizomes were purchased from Bozhou Yufangtang Biotechnology Co., Ltd. and authenticated as the rhizomes of *F. dibotrys (D. Don) Hara*, meeting the standards of the *Chinese Pharmacopoeia* (2020 edition).

Cell culture reagents included DMEM high-glucose culture medium, fetal bovine serum (FBS), and penicillin-streptomycin solution (Wuhan Pricella Biotechnology Co., Ltd., Cat. Nos.: PM150210, 164210-50, PB180120). Trypsin-EDTA (0.25%) (Thermo Fisher Scientific, United States, Cat. No.: 25200-072). The Cell Counting Kit-8 (CCK-8), crystal violet staining solution, lipid peroxidation (MDA) assay kit, Erastin, reactive oxygen species (ROS) assay kit, mitochondrial membrane potential assay kit (JC-1), BCA protein quantification assay kit, and cell membrane red fluorescence probe (Dil) (Shanghai Beyotime Biotechnology Co., Ltd., Cat. Nos.: C0038, C0121, S0131S, SC0224, S0033M, C2006, P0399S, C1036). The glutathione (GSH) assay kit (Wuhan Elabscience Biotechnology Co., Ltd., Cat. No.: E-BC-K030-M). RNA extraction, reverse transcription, and PCR amplification kits were purchased from TIANGEN Biotech (Beijing) Co.,Ltd. (Catalog Numbers:DP424, KR116, KT109). The FerroOrange ferrous ion fluorescent probe kit (Shanghai Maokang Biotechnology Co., Ltd., Cat. No.: MX4559-24UG). RIPA lysis buffer (Shanghai Wansheng Haotian Biotechnology Co., Ltd., Cat. No.: EZPS04-1). Ferrostatin-1 (Fer-1), Z-VAD-FMK, 3-Methyladenine (3-MA), Necrostatin-1 (Nec-1), and Erastin (MedChemExpress, Cat. Nos.: HY-100579, HY-16658B, HY-B1625, HY-19312, HY-15760, HY-15763). The p53, GPX4, and xCT antibodies (abcam, Cat. Nos.: ab131442, ab219592 and ab307601) ALOX15 antibodies (Hangzhou Diagbio Biotechnology Co., Ltd., Cat. No.: db11148), the β-actin antibody (Abbkine Biotechnology Co., Ltd., Cat. No.: AC028). Ki-67 polyclonal antibody and HRP-conjugated Goat Anti-Rabbit IgG (H+L) (Proteintech, Cat. Nos.: 28074-1-AP, SA00001-2).

## Methods

### Extraction and purification of FdNVs

FdNVs were extracted using ultracentrifugation. Specifically, he rhizomes of *F. dibotrys* was disrupted using ultrapure water as a solvent, and the resulting liquid was filtered. The crude filtrate underwent differential centrifugation for cellular fractionation. Initial clarification was achieved through sequential centrifugation at 1,000 × g (60 min, 4°C) followed by 10,000 × g (60 min, 4°C) to eliminate cellular suspensions, non-viable cells, particulate debris, and larger vesicles. The resulting supernatant was then sterile-filtered using a 0.45 μm pore-size membrane and subjected to ultracentrifugation at 150,000 × g (4°C, 1 h) for final particle isolation. The supernatant was discarded, and the pellet, containing FdNVs, was collected. To further purify the FdNVs, sucrose density gradient ultracentrifugation was performed (He et al., 2024). A discontinuous density gradient was prepared in polycarbonate ultracentrifuge tubes by sequentially layering sucrose solutions at increasing concentrations (8%, 30%, 45%, and 60% w/v). Following sample loading, ultracentrifugation was performed at 150,000 × g (4°C, 1 h) to achieve particle separation. The target fractions, corresponding to the interfaces between 45% and 60% sucrose layers, were carefully harvested for downstream analysis. These fractions were then diluted with an equivalent volume of PBS and subjected to centrifugation once more at 150,000 × g for 1 h. The resulting pellet was resuspended in 1 mL of PBS and either utilized immediately for subsequent experiments.

### Identification of FdNVs

#### Measurement of FdNVs particle size by NTA

Using the ZetaView PMX 110 nanoparticle tracking analyzer (Particle Metrix, Germany). The extracted and purified FdNVs were resuspended in sterile PBS to a final concentration of 1 × 10^9^ particles/mL. Gradient dilution was performed to ensure an optimal number of particles per field of view (20–100 particles), and large particle impurities were removed via filtration. The NTA instrument was set up by adjusting the laser wavelength and camera parameters, and the temperature was maintained at room temperature. A 1 mL syringe was used to slowly inject the diluted FdNVs sample into the detection chamber, ensuring uniform distribution while preventing bubble formation. Real-time visualization through a microscope was used to adjust focus and achieve a clear view of the nanovesicles. Each measurement lasted 30 s at a frame rate of approximately 25 fps, with multiple measurements conducted for accuracy. The software automatically analyzed particle trajectories and calculated the size distribution based on the Stokes-Einstein equation.

#### Observation of FdNVs morphology by TEM

To further examine the morphology of FdNVs, TEM (HT7700 model transmission electron microscope, Hitachi, Japan) analysis was performed. A sufficient amount of FdNVs solution was gently vortexed to ensure uniform dispersion. Copper grids coated with a carbon membrane (200–400 mesh) were pretreated with 30 min of UV irradiation to enhance nanovesicle adsorption. A 10 µL aliquot of the FdNVs solution was carefully pipetted onto the grid and allowed to stand for 1–2 min, enabling natural adsorption onto the grid surface, followed by gentle removal of excess liquid using filter paper. The grid was stained using 10 µL of 2% phosphotungstic acid (pH 6.8) for 30–60 s. Subsequently, any excess stain was carefully removed. The stained grid was then air-dried either in a desiccator or at room temperature for approximately 15 min to avoid contamination. Imaging was performed under an accelerating voltage of 120 kV, with appropriate magnification settings used to observe the morphology, size, and distribution of FdNVs. Multiple fields of view were captured to obtain high-quality images.

#### BCA assay for measuring the protein concentration of FdNVs

The mass concentration of FdNVs was determined based on their total protein content using the bicinchoninic acid (BCA) assay, a method commonly employed in studies involving plant-derived extracellular vesicles (Paterna et al., 2022). Following ultracentrifugation and sucrose gradient purification, FdNVs were resuspended in 1 mL PBS. An aliquot of this suspension was used to quantify protein concentration using a standard BCA protocol, with bovine serum albumin (BSA) as the standard. A calibration series was prepared by performing serial dilutions of BSA stock solution (1 mg/mL) to yield concentrations ranging from 0 to 1 mg/mL (0, 0.1, 0.2, 0.3, 0.4, 0.6, 0.8, and 1 mg/mL). The BCA working reagent was freshly prepared by combining reagents A and B at a 50:1 ratio. For the analytical procedure, 20 µL aliquots of either standards or FdNVs samples were dispensed into a 96-well plate in triplicate, followed by addition of 200 µL working reagent per well. After 30-min incubation at 37°C and subsequent cooling to ambient temperature, absorbance was measured at 562 nm using a microplate spectrophotometer. The protein concentration of the FdNV sample (expressed in mg/mL) was calculated based on the absorbance value and the BSA standard curve. This protein concentration was used as a proxy for the mass concentration of FdNVs in all subsequent *in vitro* and *in vivo* experiments. This approach allowed consistent dosing without the need for lyophilization or drying.

### *In Vitro* verification of FdNVs in inhibiting HCC cell proliferation, migration, and invasion

#### Uptake of FdNVs by HCC cells

HepG2 cell monolayers were processed through sequential washing steps using PBS following incubation. Cellular detachment was achieved through brief trypsinization (2 min), which was immediately neutralized by addition of complete growth medium. The cell suspension was then pelleted by centrifugation (350 × g, 5 min), after which the supernatant was removed and cells were reconstituted in fresh medium to a density of 3 × 10^4^ cells/mL. Aliquots (2 mL) were seeded into 6-well plates, yielding 6 × 10^4^ cells per well, and permitted to attach prior to experimental manipulation. For fluorescent labeling, FdNVs were conjugated with 10 μM Dil solution in a light-protected environment (30 min incubation). Unbound dye was removed by high-speed centrifugation (10,000 × g, 30 min). Parallel control preparations received equivalent Dil-PBS treatment. Following incubation with labeled particles, cells underwent PBS washing (×2), nuclear counterstaining with Hoechst (10 min), and additional PBS washes. Culture medium (2 mL) was replenished for 30 min temperature equilibration prior to microscopic evaluation of FdNVs cellular internalization using fluorescence imaging.

#### Cell viability assay

The cytotoxic potential of FdNVs against HCC cells was examined through CCK-8 analysis. Actively proliferating HepG2 cells were plated in 96-well microplates at 5 × 10^3^ cells/well in 100 μL complete medium. Following a 24-h attachment period, cultures were allocated into experimental cohorts: blank (medium only), untreated control, and FdNVs-treated groups with escalating concentrations. After 24-h and 48-h exposure to treatments, 10 μL CCK-8 solution was introduced to each well. Following 60-min incubation at 37°C, absorbance values were quantified at 450 nm using a microplate spectrophotometer. Relative cell viability was determined by comparing experimental groups to untreated controls, with blank values subtracted for normalization. The half-maximal inhibitory concentration (IC_50_) of FdNVs at each time point was calculated using a four-parameter logistic nonlinear regression model in GraphPad Prism 9.0. Dose–response curves were generated by plotting the logarithm of FdNVs concentration against normalized cell viability. IC_50_ values were defined as the concentration of FdNVs required to reduce cell viability to 50% relative to the control group.

#### Colony formation assay

To assess the dose-dependent inhibitory effects of FdNVs on HCC cells, exponentially growing HepG2 cells were plated in 6-well culture plates (500 cells/well). Cells were exposed to varying concentrations of FdNVs (0, 10, 25, and 50 μg/mL), representing untreated, low, medium, and high-dose treatment groups, respectively. The highest concentration was selected based on previously determined IC_50_ values from dose-response studies. Following 24-h incubation at 37°C in a humidified 5% CO_2_ atmosphere, cultures were maintained in complete growth medium for 10 days to allow colony development. Subsequently, cells were washed with PBS, fixed with methanol, and stained with 0.5% (w/v) crystal violet solution for 30 min at room temperature. After thorough rinsing and air-drying, colonies were documented by digital imaging. All experimental conditions were performed in triplicate to ensure statistical reliability.

#### EdU cell proliferation assay

To evaluate the anti-proliferative effects of FdNVs, exponentially dividing HepG2 cells were plated in 6-well plates (5 × 10^4^ cells/well) and allowed to adhere overnight. Cells were subsequently exposed to increasing FdNVs concentrations (0, 10, 25, and 50 μg/mL) for designated treatment periods. For proliferation assessment, a 20 μM EdU working solution was diluted 1:1 with culture medium to yield a final 10 μM concentration. Following 2-h incubation at 37°C, cells were fixed with 4% paraformaldehyde (15 min) and permeabilized using 0.3% Triton X-100 (10 min, RT). The Click-iT reaction was performed under light-protected conditions (30 min, RT) after thorough PBS washing (3 × 5 min). Nuclei were visualized with DAPI counterstain (10 min), followed by additional PBS washes.

#### Wound healing assay

Actively proliferating HepG2 cells were plated in 6-well culture dishes (5 × 10^4^ cells/well) and grown to complete monolayer formation. A uniform linear wound was introduced into the confluent cell layer using a sterile 200 μL pipette tip. Cells were exposed to varying concentrations of FdNVs (0, 10, 25, and 50 μg/mL). Following treatment administration, cells were maintained under standard culture conditions (37°C, 5% CO_2_). Wound closure was documented at 24-h intervals through phase-contrast microscopy, with quantitative analysis performed using ImageJ software to calculate relative migration rates.

#### Transwell invasion assay

HepG2 cell suspensions (2 × 10^4^ cells in 200 μL serum-free DMEM) were loaded into the upper chambers and were exposed to varying concentrations of FdNVs (0, 10, 25, and 50 μg/mL). The lower chambers contained 600 μL complete medium (DMEM with 20% FBS) as a chemotactic stimulus. After 24-h incubation (37°C, 5% CO_2_), non-invading cells were mechanically removed from the upper membrane surface. Invaded cells were fixed (4% paraformaldehyde, 20 min), stained (0.5% crystal violet, 10 min), and quantified microscopically. All experimental conditions were replicated three times to ensure methodological rigor.

### *In Vitro* validation of FdNVs in inhibiting HCC via ferroptosis pathway

#### Detection of Fe^2+^ levels

The study employed five treatment conditions: vehicle control (0 μg/mL FdNVs), three FdNVs concentrations (10, 25, and 50 μg/mL), and 10 μM (Yan et al., 2022) Erastin (a known ferroptosis inducer (Li et al., 2025)) as positive control. HepG2 cells (5 × 10^4^ cells/well) were cultured in 6-well plates overnight under standard conditions (37°C, 5% CO_2_) to ensure proper attachment prior to treatment exposure. For Fe^2+^ quantification, FerroOrange reagent was prepared as follows: A 1 mM stock solution was generated by dissolving 24 μg reagent in 35 μL DMSO. Working solution (1 μM) was prepared by dilution in neutral buffer. Solution was equilibrated to ambient temperature for 30 min before application. Following 24-h treatment with experimental compounds, cells were incubated with FerroOrange working solution (30 min, light-protected conditions). Intracellular ferrous iron levels were visualized using fluorescence microscopy (excitation: 488 nm; emission: 572 nm), with image analysis performed to quantify relative Fe^2+^ concentrations.

#### Detection of intracellular lipid peroxidation and ROS levels

The experimental design comprised five treatment conditions: vehicle control (0 μg/mL FdNVs), three FdNVs concentrations (10, 25, and 50 μg/mL), and 10 μM Erastin. HepG2 cells (5 × 10^4^ cells/well) were plated in 6-well plates and allowed to adhere overnight under standard culture conditions prior to 24-h compound exposure. For reactive oxygen species (ROS) detection, the fluorescent probe DCFH-DA was prepared by 1:1,000 dilution in serum-free medium, yielding a 10 μM working solution. Following treatment, cells were incubated with the probe (37°C, 20 min) and subsequently washed (3×) with serum-free medium to eliminate unincorporated dye. Fluorescence microscopy was employed to visualize intracellular ROS accumulation, with quantitative analysis performed using ImageJ software to determine relative oxidative stress levels.

#### Measurement of MDA and GSH levels

The study employed specific assay kits to evaluate malondialdehyde (MDA) and reduced glutathione (GSH) levels as biomarkers of oxidative stress. HepG2 cells were plated in 6-well culture dishes (5 × 10^4^ cells/well) and divided into five treatment cohorts: vehicle control (0 μg/mL FdNVs), three FdNVs concentrations (10, 25, and 50 μg/mL), and 10 μM Erastin. Following 24-h incubation under standard culture conditions, cellular lysates were prepared for biochemical analysis. Spectrophotometric measurements were performed using a multi-mode microplate reader with the following parameters: GSH quantification at 412 nm wavelength and MDA detection at 532 nm wavelength. The obtained optical density values were normalized to protein content and compared across treatment groups to assess treatment-induced alterations in oxidative stress parameters. All experimental procedures strictly followed the manufacturers’ protocols to ensure reproducibility.

#### Transmission electron microscopy

HepG2 cells were plated in 6-well culture plates at a density of 5 × 10^4^ cells/well and allocated into three experimental groups: untreated control, high-dose FdNVs (FdNVs-H), and Erastin-treated. Following 24-h treatment exposure, cellular specimens were processed for ultrastructural analysis through sequential preparation steps. Initial fixation was performed using ice-cold 2.5% glutaraldehyde (4°C, 2 h), followed by three 10-min washes with 0.1 M phosphate-buffered saline (PBS, pH 7.4). Secondary fixation employed 1% osmium tetroxide (1 h, light-protected conditions). A graded ethanol series (30%, 50%, 70%, 90%, and 100%) was used for cellular dehydration (10 min per concentration), followed by absolute acetone treatment. For ultramicrotomy, samples were embedded in epoxy resin and polymerized (70°C, 48 h). Ultrathin sections (70–90 nm) were collected on copper grids and double-stained with 2% uranyl acetate and lead citrate (10 min each). After thorough rinsing with distilled water and air-drying, cellular ultrastructure was examined by transmission electron microscopy, with digital images captured for comparative analysis of treatment-induced morphological alterations.

#### Mitochondrial membrane potential (MMP) assay

The cationic dye JC-1 was employed to evaluate treatment-induced alterations in mitochondrial membrane potential (ΔΨm) in HepG2 cells. Cells were cultured in 6-well plates (2 × 10^5^ cells/well) and divided into five experimental conditions: vehicle control (0 μg/mL FdNVs), three FdNVs concentrations (10, 25, and 50 μg/mL), and 10 μM Erastin as positive control, with 20-h treatment duration. For ΔΨm analysis,the JC-1 working solution was prepared according to the manufacturer’s protocol. Cells were incubated with 1 mL of the staining solution at 37°C for 20 min. The unincorporated dye was then removed by washing the cells twice with JC-1 buffer. Finally, the washed cells were maintained in 2 mL of fresh culture medium. Fluorescence microscopy visualized JC-1 aggregates (red fluorescence, high ΔΨm) and monomers (green fluorescence, depolarized mitochondria). Quantitative analysis of fluorescence intensity ratios (590 nm/530 nm) was performed using ImageJ software to determine relative changes in mitochondrial polarization status across treatment groups.

#### RT-qPCR analysis

HepG2 cells were seeded into 6-well plates and cultured in a 37°C incubator with 5% CO_2_ until they reached 70%–80% confluence. Subsequently, experimental groups were established, including FdNVs (0, 10, 25, and 50 μg/mL) and Erastin (10 μM) groups, and the cells were treated according to their respective group assignments. Upon completion of the incubation period, the culture medium was removed, and the cells were washed three times with pre-chilled PBS. Total RNA was extracted according to the reagent instructions. RNA purity and integrity were detected by A260/A280 ratio and agarose gel electrophoresis. 1 μg of RNA was taken and reverse transcribed into cDNA using a reverse transcription kit, which was used as a template for qPCR amplification reaction. The program included denaturation at 95°C for 5 min; 95°C for 10 s and 60°C for 30 s for a total of 40 cycles, and the melting curve was ramped up from 65°C to 95°C for 5 s at 0.5°C every 0.5°C to ensure that there was no non-specific amplification. Ct values were obtained and the fold expression of the target gene relative to the GAPDH gene was calculated using the 2^−ΔΔCt^ method. The primers used in this experiment were designed by Sangon Biotech Co., Ltd., Shanghai, and are listed in Table 1.

**TABLE 1:** Primer sequences.

Gene name	Primer sequence (5′∼3′)	Product length/bp
*GPX4*	F: CCC​GAT​ACG​CTG​AGT​GTG​GTT​TG	82
	R: TCT​TCG​TTA​CTC​CCT​GGC​TCC​TG	
*SLC7A11*	F: GGA​TTG​GCT​TCG​TCA​TCA​CTC​TG	97
	R: TTC​TCC​GAC​ATT​ATT​CTA​AAC​CAC​CTG	
*P53*	F: GAGGAGCCGCAGTCAGATCC R: CCAGGAGCAGAGACACCATC	120
*ALOX15*	F: ACT​TGA​TGG​CTG​AGG​TCA​TTG​TTG	101
	R: TTC​CAG​GGT​GTA​TCG​CAG​GTG	
*GAPDH*	F: ACA​CCC​ACT​CCT​CCA​CCT​TTG	112
	R: TCC​ACC​ACC​CTG​TTG​CTG​TAG	

### *In Vivo* verification of FdNVs in restricting HCC xenograft growth

#### Animal grouping, model establishment, and drug administration

Male BALB/c nude mice (4–5 weeks old) were randomly allocated into five treatment cohorts (n = 5 per group): vehicle control, low-dose FdNVs (FdNVs-L), medium-dose FdNVs (FdNVs-M), high-dose FdNVs (FdNVs-H) and Sorafenib positive control. Tumor xenografts were established through subcutaneous inoculation of 5 × 10^6^ HepG2 cells (suspended in 100 μL serum-free DMEM) into the right dorsal flank. Treatment initiation occurred upon reaching palpable tumor volumes (approximately 50–100 mm^3^). Throughout the experimental period, animals were maintained under standard housing conditions with *ad libitum* access to sterilized food and water. The FdNVs used in this study were equivalent to 100 times the clinical equivalent dose of *F. dibotrys* (based on the amount of crude drug). The experiments employed the body surface area-based equivalent dose coefficient method for dose conversion, referencing the human and animal equivalent dose ratio table in “Pharmacological Experimental Methodology” to convert the human dose to the equivalent mouse dose. For the FdNVs groups, the low dose was set at 15.38 mg/kg/day via gavage, the medium dose at 30.75 mg/kg/day via gavage, and the high dose at 61.5 mg/kg/day via gavage. For the Sorafenib group,a dose of 30 mg/kg was administered via gavage every other day (Yamamoto et al., 2015).

#### Body weight and tumor volume measurement

During the drug treatment period, body weight was measured every 3 days, and relevant data were recorded to evaluate the effects of FdNVs on mouse growth. After 14 days of intervention, mice were euthanized in accordance with ethical guidelines, and tumors were excised and photographed. Tumor length and width were measured using calipers, and tumor volume was calculated using the formula: $V=\frac{a\times b^{2}}{2}$ , where a represents the maximum tumor length and b represents the perpendicular tumor width.

#### Hematoxylin and eosin (H&E) staining

Tumor tissues from each experimental group were fixed in 4% paraformaldehyde for 24 h, then dehydrated through a graded ethanol series, cleared in xylene, and embedded in paraffin before being sectioned at 4 μm thickness and mounted on slides for drying at 37°C, after which the sections were deparaffinized in xylene and rehydrated through a descending ethanol series (100%, 95%, 80%, and 70%) followed by distilled water rinsing, then stained with hematoxylin for 5 min and rinsed with running water before differentiation in 1% hydrochloric acid-ethanol solution and counterstaining with eosin for 1–2 min, then washed again and dehydrated through an ascending ethanol series, cleared in xylene, and mounted with neutral balsam for microscopic examination of histopathological changes using light microscopy with image capture.

#### Immunohistochemical (IHC) staining

Tumor specimens were initially fixed in 4% paraformaldehyde (24 h), then sequentially processed through ethanol dehydration, xylene clearing, and paraffin embedding before sectioning at 4 μm thickness. Mounted sections were heat-treated at 60°C (1 h) to enhance tissue adhesion, followed by deparaffinization in xylene and graded ethanol rehydration (100%, 95%, 85%, 75%). Antigen retrieval was conducted in sodium citrate buffer (pH 6.0) using microwave irradiation, with subsequent cooling and PBS rinsing. For immunostaining, endogenous peroxidase activity was quenched with 3% H_2_O_2_ (10 min), followed by protein blocking using 5% BSA (30 min). Sections were incubated with anti-Ki67 primary antibody (4°C, overnight), washed, then treated with HRP-conjugated secondary antibody (37°C, 30 min). Chromogenic development employed DAB substrate, with reaction termination upon optimal staining intensity. Counterstaining with hematoxylin (1 min) preceded final dehydration and mounting. Quantitative analysis of Ki67-positive regions was performed using light microscopy with digital image analysis.

#### Western blot analysis

To optimize the extraction and analysis of proteins from both cell cultures and tissue samples, a unified protocol was implemented. Following treatment, cells in 6-well plates or weighed tissue samples were lysed using a buffer containing protease and phosphatase inhibitors. For cell cultures, lysis was achieved by scraping, while tissues were homogenized at a buffer-to-tissue weight ratio of 1: 10. The lysates were incubated on ice for 30 min, with vortexing every 10 min to ensure complete lysis. Subsequently, the lysates were centrifuged at 12,000 × g for 15 min at 4°C, and the supernatant was carefully collected and stored at-20°C for further analysis. The protein concentration was measured using the BCA assay, and an appropriate volume of protein sample was combined with 5× SDS-PAGE loading buffer and denatured at 95°C for 5 min. Proteins were separated by SDS-PAGE using a 10% gel run at 120 V until the dye front reached the bottom of the gel. The proteins were then transferred to a PVDF membrane using a semi-dry transfer system at 400 mA for 30 min. The membrane was blocked with 5% non-fat milk in TBS-T (TBS with 0.1% Tween-20) for 1 h at room temperature. Primary antibodies (p53 (1:1,000), xCT (1:4,000), GPX4 (1:4,000), ALOX15 (1:1,000), and β-actin (1:10,000)) were diluted in 5% non-fat milk in TBS-T and incubated with the membrane overnight at 4°C. The membrane was washed three times with TBS-T (10 min each) to remove unbound primary antibodies. HRP-conjugated secondary antibodies were applied and incubated for 1 h at room temperature, followed by three additional washes with TBS-T (10 min each). Protein bands were detected using an ECL substrate and visualized with a chemiluminescent imaging system. This protocol ensures consistent and reproducible results for both cell and tissue samples, facilitating accurate protein analysis.

#### Statistical analysis

All statistical analyses were conducted with SPSS version 25.0 (IBM Corp.), with continuous variables presented as mean ± SEM. The selection of appropriate statistical tests was based on data distribution characteristics and variance homogeneity, assessed through Shapiro-Wilk and Levene’s tests, respectively. For parametric data meeting both normality and homogeneity assumptions, intergroup comparisons were performed using one-way analysis of variance (ANOVA), with Tukey’s honestly significant difference (HSD) test for multiple comparisons. When normality was satisfied but variance homogeneity was violated, Welch’s ANOVA with Games-Howell *post hoc* testing was implemented. Non-parametric datasets were analyzed using the Kruskal-Wallis test, followed by Dunn’s procedure for pairwise comparisons. Throughout all analyses, a two-tailed probability value below 0.05 was considered indicative of statistical significance.

## Results

### Isolation, extraction, and characterization of FdNVs

**FIGURE 1:**
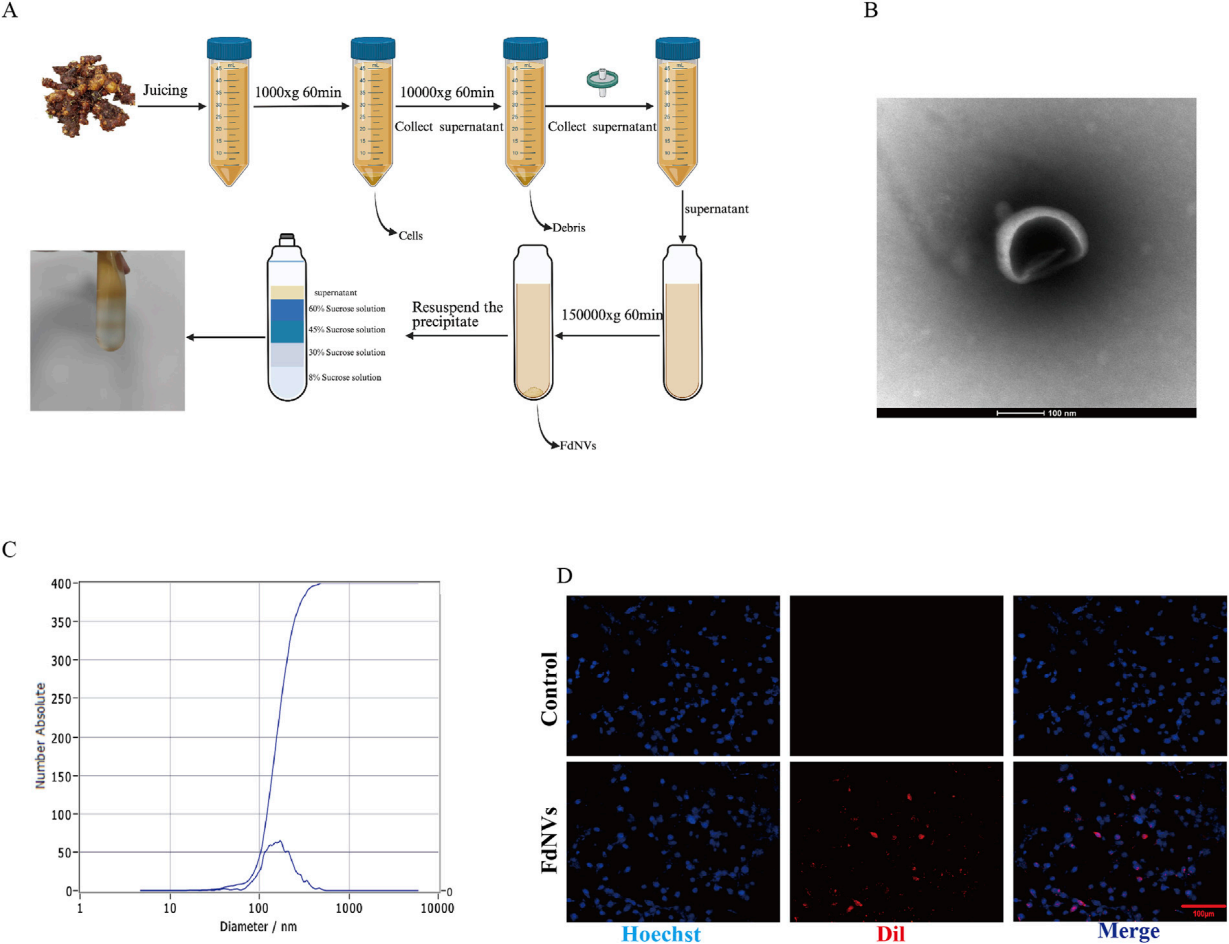
Characterization of FdNVs. **(A)** Schematic diagram of FdNVs extraction. **(B)** Morphology of FdNVs observed by TEM after ultracentrifugation. **(C)** Size distribution of FdNVs measured by NTA. **(D)** Uptake of FdNVs by HepG2 Cells.

FdNVs were successfully isolated and extracted using ultracentrifugation (Figure 1A). TEM revealed that FdNVs exhibited a characteristic vesicle-like morphology with a diameter ranging between 50 and 200 nm (Figure 1B). Additionally, NTA was performed to measure the size distribution and dispersion of FdNVs, showing that the hydrodynamic diameter of FdNVs was 168.7 nm, with a uniform size distribution, accounting for 100% of the particles (Figure 1C). BCA protein quantification of purified FdNVs indicated a protein concentration of 0.2074 mg/mL.

### FdNVs uptake by HepG2 cells

To assess the internalization capacity of FdNVs within HepG2 cells, Dil red fluorescent probe was used to label FdNVs. Dil is a widely utilized fluorescent dye in cellular uptake studies, capable of emitting a stable red fluorescence signal once internalized by cells. In this experiment, Dil-labeled FdNVs were co-incubated with HepG2 cells, and their uptake was observed under a fluorescence microscope. The results demonstrated that in the FdNVs-treated group, intense red fluorescence signals were observed within the cytoplasm of HepG2 cells, indicating that the labeled FdNVs were successfully internalized and accumulated within the cells. In contrast, no red fluorescence signal was observed in the PBS control group, confirming that HepG2 cells did not take up Dil-labeled FdNVs in the absence of treatment. This result validates the specificity of FdNVs uptake, as cells without FdNVs exposure exhibited no background fluorescence signal (Figure 1D).

### Effect of different concentrations of FdNVs on HepG2 cell viability

**FIGURE 2:**
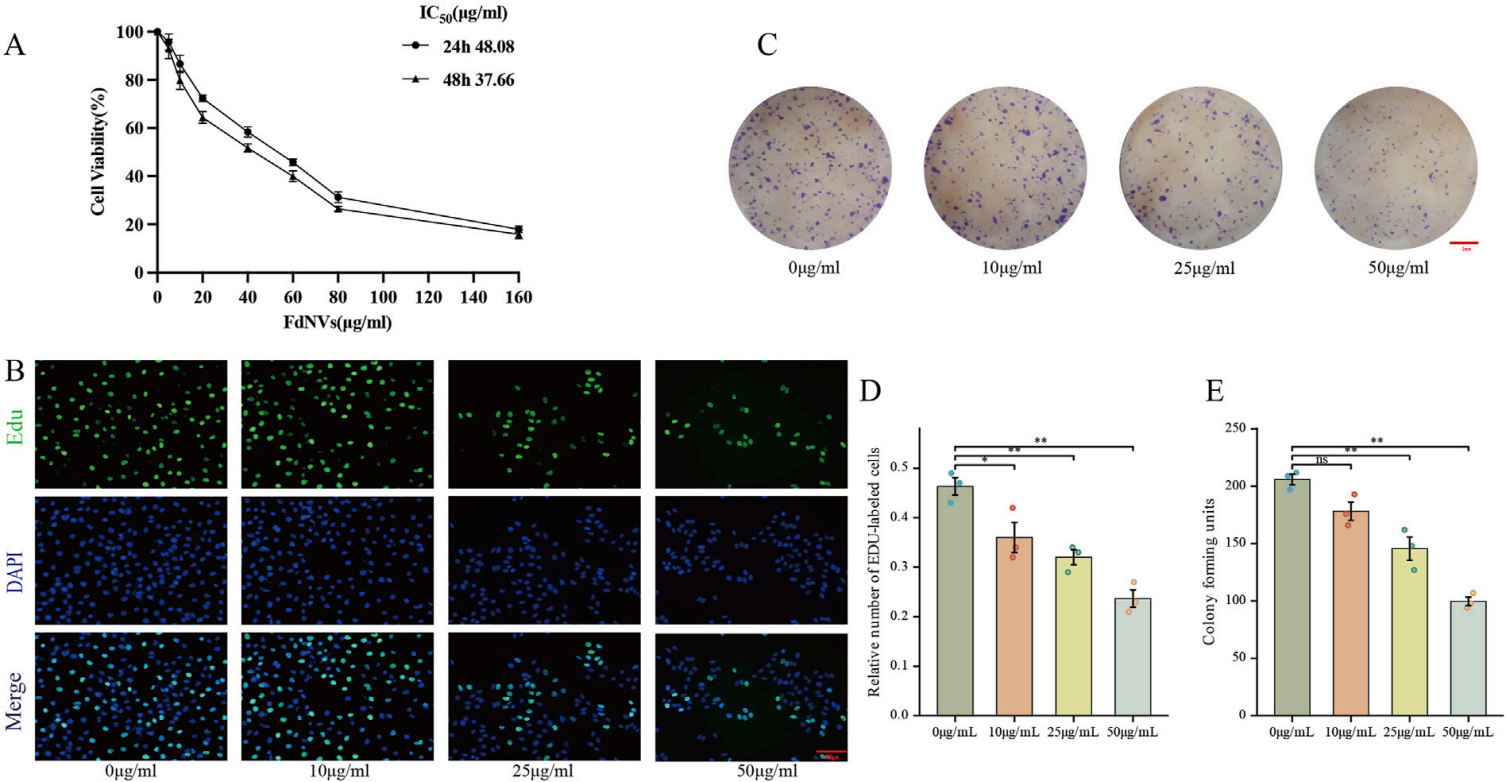
Effects of FdNVs on HepG2 Cell Proliferation. **(A)** FdNVs reduced HepG2 cell viability. CCK-8 assay results showing cell viability after treatment with different concentrations of FdNVs for 24 or 48 h **(B)** EdU assay for cell proliferation. EdU-positive proliferating cells are shown in green, while DAPI-labeled live cells are shown in blue. **(C)** FdNVs inhibited HepG2 colony growth. **(D)** Quantification of relative EdU-positive cell numbers. **(E)** Statistical analysis of colony-forming units. Data are presented as mean ± SEM (n = 3). Statistical significance was assessed relative to the 0 μg/mL FdNVs group: *p < 0.05, **p < 0.01, ns (not significant, *p* > 0.05).

The impact of FdNVs on the viability of HepG2 cells at varying concentrations and durations (24 and 48 h) was assessed using the CCK-8 assay. As shown in Figure 2A, FdNVs significantly inhibited cell viability in a dose- and time-dependent manner. The calculated IC_50_ values were 48.08 μg/mL at 24 h and 37.66 μg/mL at 48 h, indicating enhanced cytotoxicity with prolonged exposure. Notably, although 100 μg/mL exerted the strongest cytotoxic effect, it led to excessive cell death and loss of cell adhesion, resulting in cell detachment and suspension. These conditions were unsuitable for subsequent phenotypic assays (e.g., proliferation, migration, invasion), which require adherent cells for accurate observation and quantification. Based on these results, the 50 μg/mL dose—close to the 24-h IC_50_ value—was included as a high-dose group in all subsequent experiments. To better characterize the dose–response relationship, we systematically employed a gradient design using 0, 10, 25, and 50 μg/mL concentrations in all *in vitro* assays. This approach ensured comprehensive evaluation of both low and high doses, allowing mechanistic insights into the concentration-dependent effects of FdNVs on ferroptosis induction and mitochondrial dysfunction in HepG2 cells.

### FdNVs inhibit HepG2 cell proliferation

The anti-neoplastic activity of FdNVs was evaluated through complementary proliferation assays in HepG2 cells. Quantitative analysis of DNA replication via EdU incorporation revealed marked proliferative activity in untreated controls (0 μg/mL), which showed progressive attenuation with increasing FdNVs concentrations (10–50 μg/mL). Notably, the highest concentration (50 μg/mL) induced maximal suppression of DNA synthesis (*p* < 0.01 vs. control, Figures 2B,D). Parallel assessment of clonogenic potential demonstrated significant reduction in both colony number and size following FdNVs exposure (Figures 2C,E). While control cultures formed abundant colonies, FdNVs-treated cells exhibited dose-dependent inhibition, with the 50 μg/mL group displaying only residual colony-forming capacity (*p* < 0.01). These findings demonstrate concentration-dependent growth inhibition, potentially mediated through cell cycle modulation or apoptotic induction. The concordant results from short-term (EdU) and long-term (clonogenic) proliferation assays substantiate FdNVs’ therapeutic potential against HCC.

### FdNVs suppress HepG2 cell migration and invasion

**FIGURE 3:**
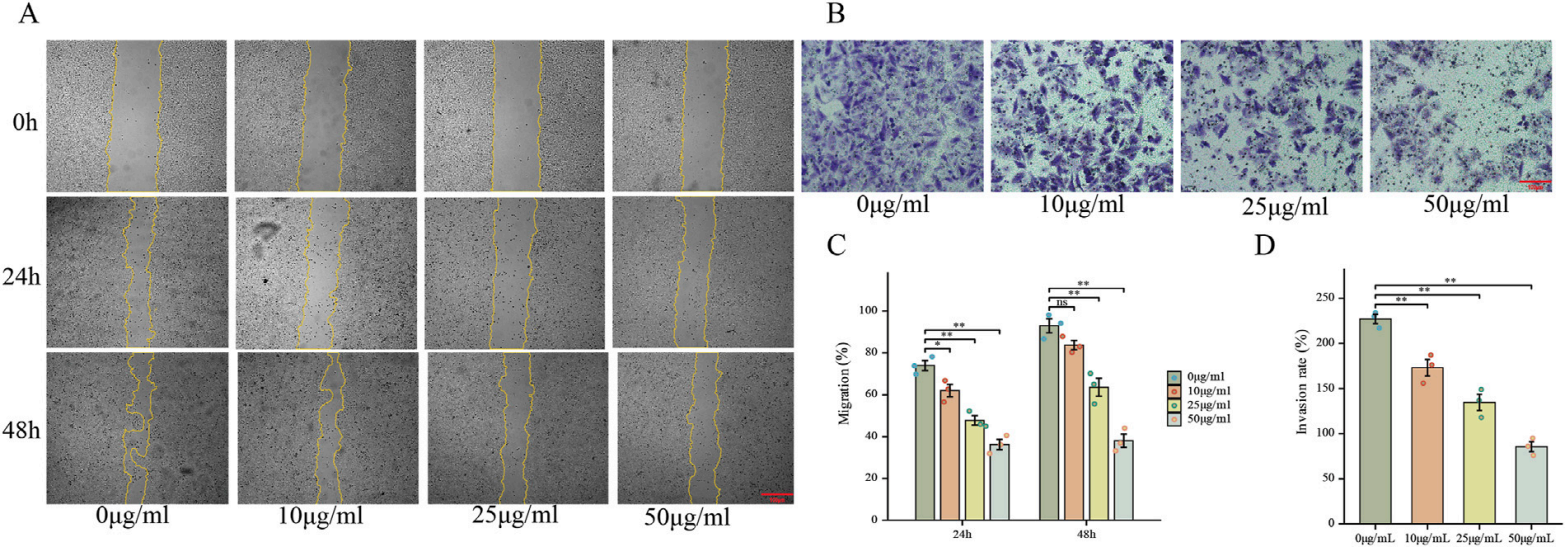
FdNVs Inhibit HepG2 Cell Migration and Invasion. **(A)** Wound healing assay showing the effect of FdNVs on HepG2 cell migration. Cells were incubated with 0, 10, 25, and 50 μg/mL FdNVs, and images were taken at 0, 24, and 48 h. Yellow lines outline the wound edges. **(B)** Transwell invasion assay (×100) assessing the invasive ability of HepG2 cells treated with FdNVs for 24 h. **(C)** Quantification of migration expressed as the percentage of the wound area covered by cells over time, with 0% corresponding to the initial wound area at 0 h. **(D)** Quantification of relative invasion rate of HepG2 cells across the membrane. Data are presented as mean ± SEM (n = 3). Statistical significance was assessed relative to the 0 μg/mL FdNVs group: *p < 0.05, **p < 0.01, ns (not significant, *p* > 0.05).

To further explore the effects of FdNVs on the migration and invasion capabilities of HepG2 cells, wound healing and Transwell invasion assays were performed. The wound healing assay simulated cell migration ability by observing scratch closure. In the 0 μg/mL group, the wound area significantly reduced after 24 and 48 h, indicating strong migratory activity. However, as the FdNVs concentration increased, scratch closure was progressively inhibited, with the 50 μg/mL group showing the least wound closure (*p* < 0.01, Figures 3A,C). The Transwell invasion assay was used to assess the ability of HepG2 cells to penetrate Matrigel-coated membranes. Compared with the 0 μg/mL group, FdNVs treatment led to a significant reduction in the number of cells that successfully invaded through the membrane, with the 50 μg/mL group showing only a minimal number of invasive cells (*p* < 0.01, Figures 3B,D). These findings suggest that FdNVs effectively inhibit HepG2 cell migration and invasion in a dose-dependent manner.

### FdNVs induce ferroptosis in HepG2 cells

**FIGURE 4:**
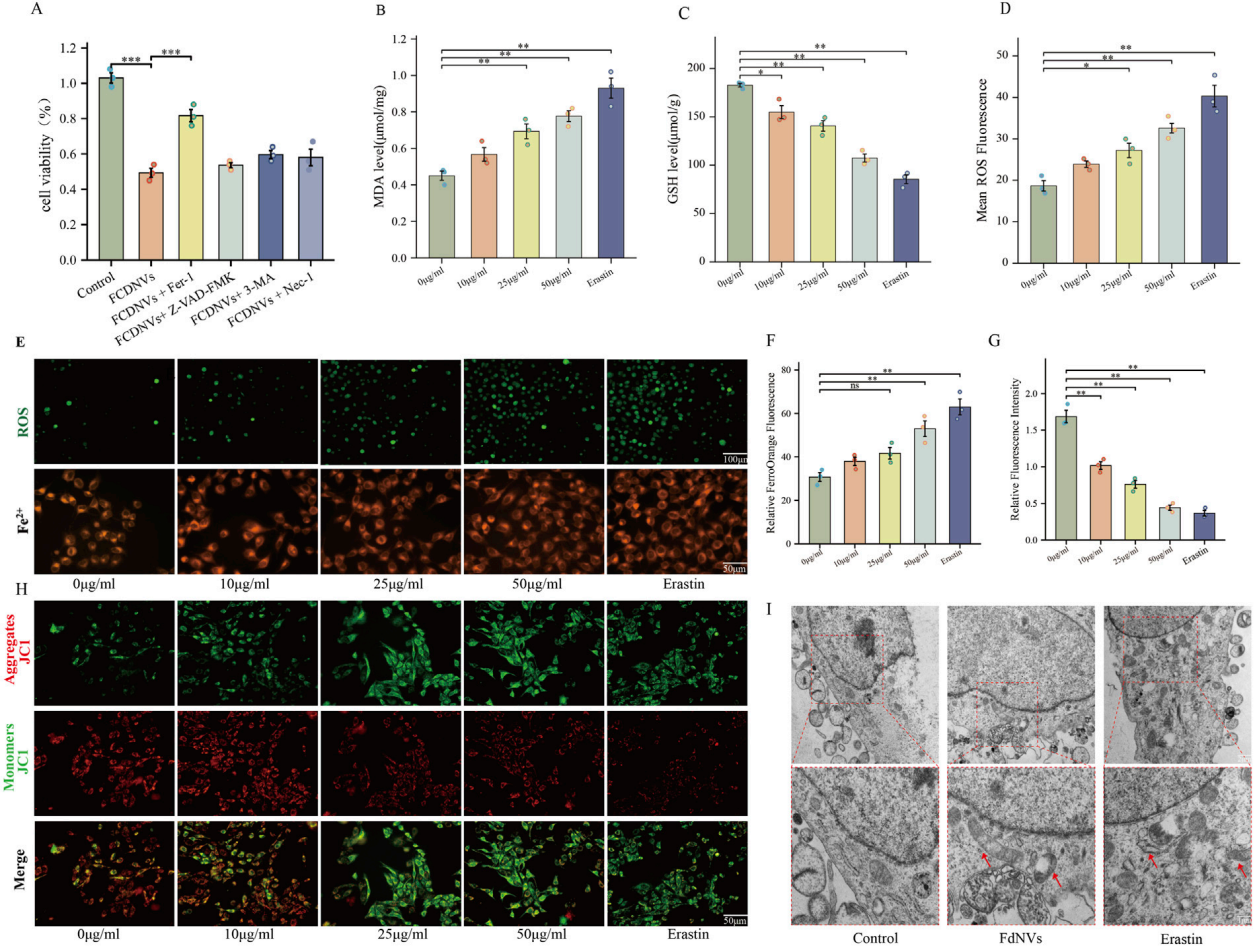
FdNVs Induce Ferroptosis in HepG2 Cells. **(A)** CCK-8 assay assessing cell viability under treatment with FdNVs (50 μg/mL) in the presence or absence of Fer-1, Z-VAD-FMK, 3-MA, and Nec-1. **(B)** MDA levels in HepG2 cells treated with different concentrations of FdNVs (0, 10, 25, 50 μg/mL) and Erastin (10 μM), measured using an MDA assay kit. **(C)** GSH levels in HepG2 cells treated with different concentrations of FdNVs (0, 10, 25, 50 μg/mL) and Erastin (10 μM), measured using a GSH assay kit. **(D)** Quantification of intracellular ROS levels in HepG2 cells treated with FdNVs, expressed as relative fluorescence intensity. **(E)** Detection of Fe^2+^ and ROS levels in HepG2 cells treated with FdNVs using FerroOrange and DCFH-DA fluorescence probes, respectively. **(F)** Quantification of FerroOrange fluorescence intensity, indicating intracellular Fe^2+^ levels. **(G)** Relative JC-1 aggregate/monomer fluorescence ratio, reflecting MMP. **(H)** JC-1 fluorescence staining showing MMP changes in HepG2 cells under different treatments. **(I)** TEM images of mitochondrial ultrastructure in HepG2 cells, highlighting ferroptosis-associated morphological changes, including mitochondrial shrinkage, cristae loss, and increased membrane density. Data are presented as mean ± SEM (n = 3). Statistical significance was assessed relative to the 0 μg/mL FdNVs group: *p < 0.05, **p < 0.01, ns (not significant, *p* > 0.05).

To identify the specific type of cell death triggered by FdNVs within a 24-h period, HepG2 cells were exposed to 50 μg/mL FdNVs, either in the presence or absence of various cell death inhibitors. The CCK-8 assay was used to assess cell viability in the following groups: Control, 50 μg/mL FdNVs, and FdNVs combined with apoptosis inhibitor (Z-VAD-FMK, 20 μM), Fer-1 (1 μM), 3-MA (2 mM), and Nec-1 (50 μM) (Yang et al., 2025). The results showed that cell viability in the FdNVs group was significantly lower than in the Control group (*p* < 0.01), confirming the inhibitory effect of FdNVs on HepG2 cells. Notably, the ferroptosis inhibitor (Fer-1) significantly restored cell viability (*p* < 0.01) compared to the FdNVs-only group, suggesting that ferroptosis plays a key role in FdNVs-induced cell death. In contrast, cell viability was not significantly improved in the apoptosis (Z-VAD-FMK), autophagy (3-MA), and necroptosis (Nec-1) inhibitor-treated groups, indicating that these pathways do not significantly contribute to FdNVs-induced cell death (Figure 4A). These findings suggest that FdNVs primarily induce ferroptosis in HepG2 cells rather than other forms of programmed cell death. To further investigate the ferroptotic effects of FdNVs, intracellular ROS levels, Fe^2+^ concentration, MDA content, and GSH levels were analyzed. Additionally, MMP was assessed using JC-1 fluorescence probes, and TEM was used to examine ultrastructural changes in mitochondria across treatment groups.

Detection of ROS levels in HepG2 cells via fluorescence indicated that the intensity of green fluorescence rose in a dose-dependent manner as the concentration of FdNVs increased. In the 50 μg/mL FdNVs group, ROS accumulation was comparable to that of the Erastin-treated positive control group, indicating significant oxidative stress (*p* < 0.01, Figures 4D–F). A similar trend was observed in Fe^2+^ fluorescence intensity, further confirming that FdNVs induce ferroptosis by promoting ROS accumulation and iron overload.

Compared to the Control group, the levels of MDA, a marker of lipid peroxidation, were significantly elevated in the FdNVs-H and Erastin groups (*p* < 0.01, Figure 4B). Conversely, GSH levels, a key ferroptosis suppressor, were markedly decreased in the 50 μg/mL FdNVs group, reaching levels comparable to the Erastin group (*p* < 0.01, Figure 4C). These findings suggest that FdNVs promote iron accumulation and enhance lipid peroxidation, ultimately triggering ferroptosis.

The JC-1 assay was used to assess MMP, a crucial indicator of mitochondrial function. The Control group exhibited dominant red fluorescence with the highest red/green fluorescence ratio, indicating normal MMP. In the FdNVs-L and FdNVs-M groups, a slight increase in green fluorescence was observed, suggesting mild MMP reduction. In the FdNVs-H group, green fluorescence was significantly enhanced, and the red/green ratio was markedly decreased, indicating substantial mitochondrial dysfunction. The Erastin group displayed the most intense green fluorescence and the lowest red/green ratio, signifying complete MMP loss. These results demonstrate that FdNVs induce dose-dependent mitochondrial membrane potential disruption, which may contribute to ferroptosis by impairing mitochondrial function (*p* < 0.01, Figures 4G,H).

TEM further confirmed ferroptosis-associated mitochondrial damage. In the Control group, HepG2 cells exhibited intact mitochondrial structures with well-defined cristae and a regular arrangement. However, in both the FdNVs-treated and Erastin groups, mitochondria displayed typical ferroptotic features, including cristae loss, membrane shrinkage, increased membrane density, and vacuole-like structures within the mitochondrial matrix. In severe cases, outer membrane rupture and leakage of mitochondrial contents were observed. These ultrastructural changes provide further evidence that FdNVs disrupt mitochondrial function and induce ferroptosis in HepG2 cells (Figure 4I).

### FdNVs regulate the p53/xCT/GPX4 signaling axis to induce ferroptosis in HepG2 cells

**FIGURE 5:**
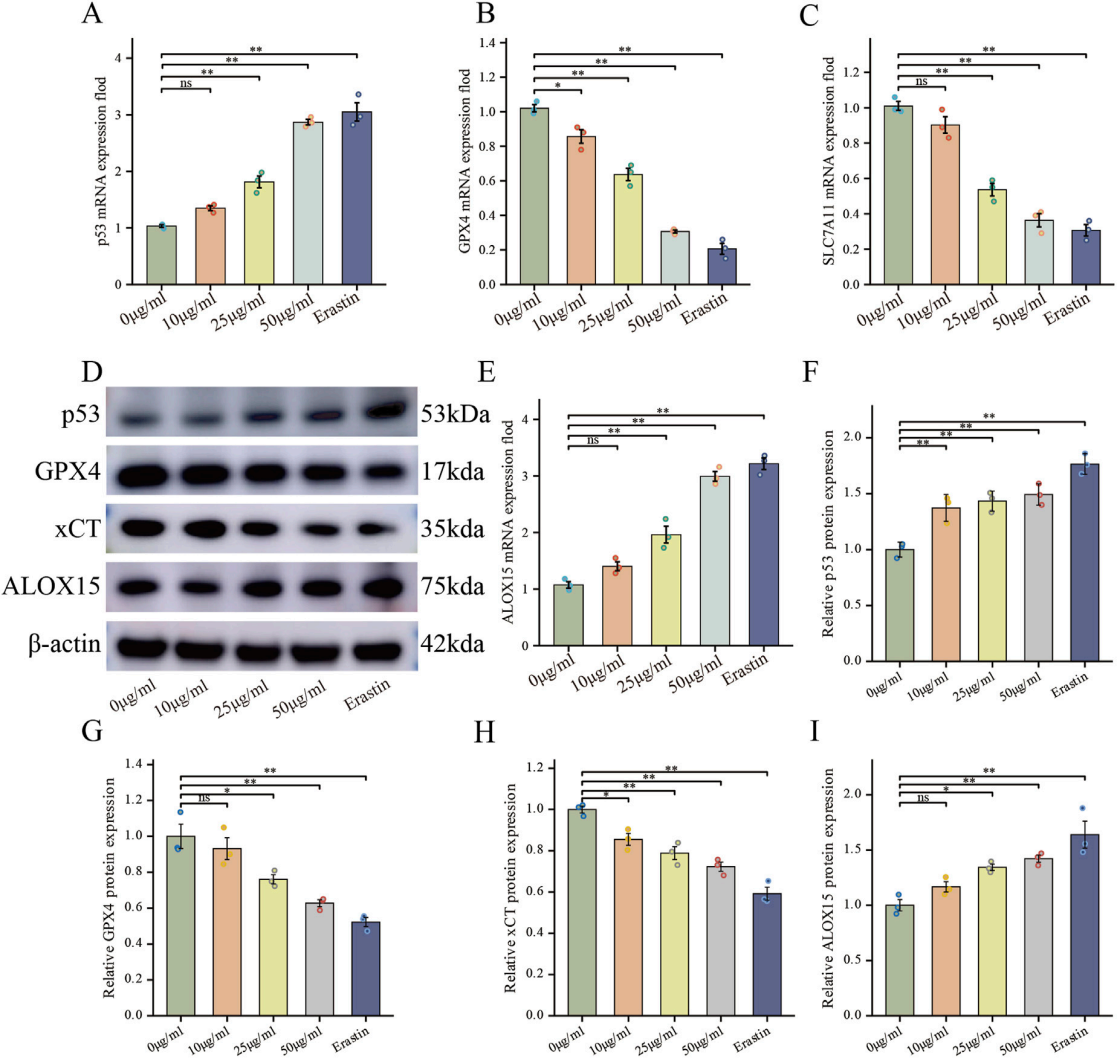
FdNVs Regulate the p53/xCT/GPX4 Signaling Axis to Induce Ferroptosis in HepG2 Cells. **(A–C,E)** RT-qPCR analysis of *p53*, *GPX4*, *SLC7A11*, and *ALOX15* mRNA expression levels in HepG2 cells treated with different concentrations of FdNVs and Erastin. **(D)** Western blotting analysis of ferroptosis-related biomarkers p53, GPX4, xCT, and ALOX15 protein expression levels under different FdNVs and Erastin treatments. **(F–I)** Quantification of relative protein expression levels of p53, GPX4, xCT, and ALOX15, normalized to β-actin. Data are presented as mean ± SEM (n = 3). Statistical significance was assessed relative to the 0 μg/mL FdNVs group: *p < 0.05, **p < 0.01, ns (not significant, *p* > 0.05).

To elucidate the molecular mechanism underlying FdNVs-induced ferroptosis, we specifically focused on the p53/xCT/GPX4 signaling axis—a well-established regulatory pathway in ferroptosis modulation (Stockwell et al., 2017). HepG2 cells were treated with increasing concentrations of FdNVs (10, 25, and 50 μg/mL), and the expression levels of key pathway components were evaluated at both the mRNA and protein levels using RT-qPCR and Western blotting, respectively. RT-qPCR analysis revealed that, relative to the Control group, treatment with FdNVs at concentrations of 25 μg/mL and 50 μg/mL, as well as with Erastin, significantly increased the mRNA expression of p53 and ALOX15, while concurrently decreasing the mRNA expression of SLC7A11 and GPX4. Western blotting analysis further confirmed these findings at the protein level (Figure 5). Upon FdNVs treatment, the expression of p53 was progressively upregulated in a dose-dependent manner, highlighting its key regulatory role in ferroptosis. Meanwhile, the ferroptosis inhibitors xCT and GPX4 were significantly downregulated, suggesting that FdNVs inhibit xCT-mediated cystine uptake, thereby impairing GPX4-dependent reduction of lipid peroxides and exacerbating oxidative damage. In contrast, ALOX15 expression was markedly upregulated, facilitating polyunsaturated fatty acid (PUFA) oxidation and further promoting lipid peroxidation. Given that p53 is known to transcriptionally repress SLC7A11 and indirectly downregulate GPX4 by depleting glutathione, the observed expression changes support the conclusion that FdNVs induce ferroptosis via the p53/xCT/GPX4 axis. Together, these data confirm that the ferroptotic effect of FdNVs in HepG2 cells involves activation of this classical signaling pathway.

### Effects of FdNVs on HCC xenografts in nude mice

To evaluate the antitumor effects of FdNVs in a subcutaneous HCC xenograft model, this study comprehensively assessed body weight changes, tumor volume, HE staining, Ki67 expression, ROS levels, TUNEL assay, and ferroptosis-related protein expression via Western blotting.

**FIGURE 6:**
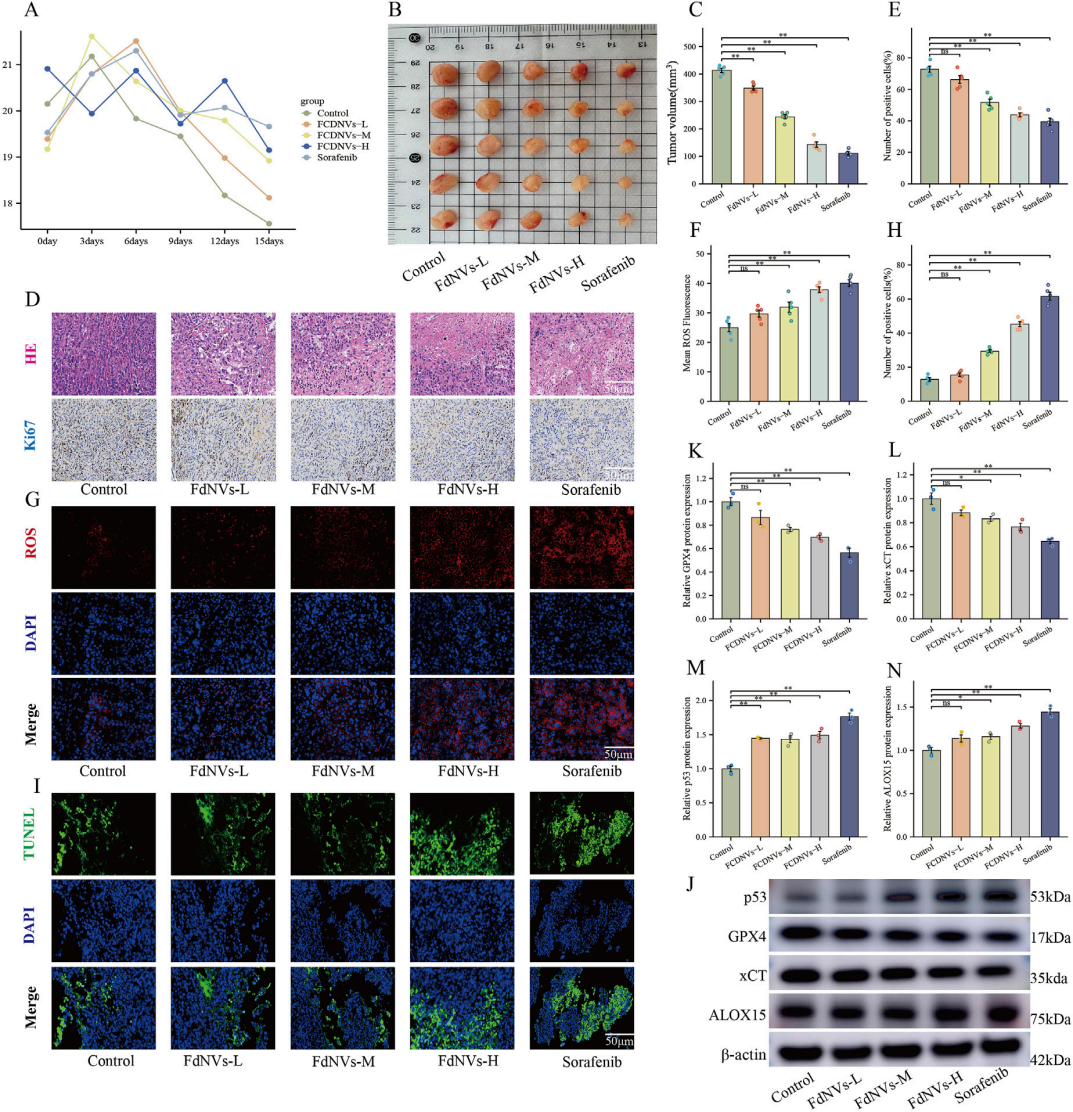
FdNVs Inhibit Tumor Growth and Promote Ferroptosis *In Vivo*. **(A)** Body weight monitoring of nude mice treated with different concentrations of FdNVs. **(B)** Representative tumor images from each treatment group. **(C)** Tumor volume analysis showing significant reduction in tumor size following FdNVs treatment in a dose-dependent manner. **(D)** Histopathological examination via HE staining and Ki67 immunohistochemical (IHC) staining to assess tumor proliferation. **(E)** Quantification of Ki67-positive cells, indicating a significant reduction in cell proliferation upon FdNVs treatment. **(F)** Quantification of ROS fluorescence intensity, demonstrating increased ROS levels in FdNVs-treated tumors. **(G)** ROS fluorescence staining showing elevated peroxide accumulation in tumor tissues. **(H)** Quantification of TUNEL-positive apoptotic cells, confirming enhanced apoptosis in the FdNVs-treated groups. **(I)** TUNEL staining images showing apoptotic cells in tumor tissues across different treatment groups. **(J)** Western blot analysis of ferroptosis-related markers GPX4, xCT, p53, and ALOX15 under different FdNVs and Sorafenib treatments. **(K–N)** Quantification of GPX4, xCT, p53, and ALOX15 protein expression levels, demonstrating ferroptosis activation in FdNVs-treated tumors. Data are presented as mean ± SEM (n = 5). Statistical significance was assessed relative to the Control group: *p < 0.05, **p < 0.01, ns (not significant, *p* > 0.05).

Throughout the 14-day treatment period, no mortality or abnormal behaviors were observed in any group. Serial measurements revealed no significant weight loss in nude mice across the various FdNVs treatment groups compared with the control group, indicating general tolerability (Figure 6A). To further evaluate the potential *in vivo* toxicity of FdNVs, major organs including the heart, liver, lungs, and kidneys were collected at the end of the experiment for histopathological examination. H&E staining results (Supplementary Figure S1) revealed normal tissue architecture in all examined organs. No signs of tissue damage, inflammatory infiltration, or necrosis were detected, confirming the absence of observable acute or subacute toxicity. These findings collectively demonstrate that FdNVs, within the tested dose range, exhibit a favorable safety profile *in vivo* and are well-tolerated by nude mice.

Meanwhile, tumor volume measurements revealed that FdNVs significantly inhibited tumor growth in a dose-dependent manner. Compared with the Control group, tumors in the FdNVs-treated groups were markedly smaller, with the FdNVs-H group exhibiting the most pronounced tumor reduction, achieving an antitumor effect comparable to that of Sorafenib (*p* < 0.01, Figures 6B,C).

HE staining further demonstrated the antitumor effect of FdNVs. In the Control group, tumor cells were densely arranged with frequent mitotic figures, indicative of high proliferative activity. In contrast, the FdNVs-treated groups exhibited extensive necrotic areas, with the extent of necrosis increasing with FdNVs dosage. The FdNVs-H group and Sorafenib group exhibited the largest necrotic regions, indicating that FdNVs effectively inhibit tumor growth. Ki67 IHC staining was performed to assess cell proliferation in tumor tissues. The Control group exhibited a high percentage of Ki67-positive cells, reflecting strong proliferative activity. However, in the FdNVs-treated groups, Ki67 expression was significantly reduced in a dose-dependent manner. Notably, Ki67 positivity in the FdNVs-H group was comparable to that of the Sorafenib group, confirming that FdNVs significantly suppress tumor cell proliferation (*p* < 0.01, Figures 6D,E).

Intracellular ROS levels in tumor tissues were evaluated using fluorescence staining. Compared with the Control group, FdNVs treatment resulted in a significant increase in ROS levels, with fluorescence intensity increasing in a dose-dependent manner. The FdNVs-H group exhibited the highest ROS accumulation, comparable to the Sorafenib group. These findings suggest that FdNVs may promote ferroptosis by inducing ROS accumulation (*p* < 0.01, Figures 6F,G).

TUNEL staining was conducted to assess apoptotic cell levels in tumor tissues. The Control group exhibited a low number of TUNEL-positive apoptotic cells, whereas the FdNVs-treated groups showed a significant increase in apoptotic cell numbers, with fluorescence intensity increasing in a dose-dependent manner. The highest number of apoptotic cells was observed in the FdNVs-H group, indicating that FdNVs significantly promote tumor cell apoptosis (*p* < 0.01, Figures 6H,I).

Western blotting analysis of ferroptosis-related protein expression in tumor tissues revealed that p53 expression increased progressively with higher FdNVs concentrations, while the expression levels of ferroptosis inhibitors xCT and GPX4 were significantly decreased. Additionally, the expression of ALOX15, a lipid oxidation-promoting enzyme, was significantly upregulated in the FdNVs-H group (*p* < 0.01, Figures 6J–N). Collectively, these findings demonstrate that FdNVs effectively suppress the growth of subcutaneous HCC xenografts in nude mice, with their mechanism likely involving ferroptosis induction. Specifically, FdNVs exert their antitumor effect by upregulating p53, downregulating xCT and GPX4, and activating ALOX15, thereby disrupting the cellular antioxidant defense system and inducing ferroptotic cell death in HepG2 cells.

## Discussion

HCC represents 75%–85% of primary hepatic malignancies, positioning it as the dominant histologic subtype among liver cancers (Shek et al., 2021). Contemporary epidemiological registries reveal dismal prognoses, with advanced-stage patients exhibiting a 5-year survival probability of merely 3% (SEER database) and median overall survival limited to 23–33 months according to multinational cohort analyses (Bray et al., 2024). The main treatment strategies for HCC include surgical resection, liver transplantation, local ablation therapy, transarterial embolization, and systemic therapy (Liu et al., 2025). In recent years, immune-combination targeted therapy (e.g., atelizumab + bevacizumab) has become the first-line treatment option for advanced HCC, significantly improving survival. However, the treatment of advanced HCC still faces many challenges, and there is an urgent need to further improve the survival and quality of life of patients (Xue et al., 2025). HCC is a highly prevalent, lethal, and recurrent malignant tumor around the world, and despite advances in diagnosis and treatment, insufficient early screening and diagnosis, limited therapeutic options, and imperfect multidisciplinary diagnostic and treatment modalities still remain clinical challenges (Qin et al., 2023; Hwang et al., 2025). Therefore, developing novel therapeutic agents with both high safety and potent antitumor activity remains an urgent priority in HCC treatment.

PDENs are extracellular vesicles secreted by plant cells, typically 30–200 nm in diameter, and are rich in lipids, proteins, RNA, and secondary metabolites (e.g., polyphenols and flavonoids). These vesicles are released via endosomal pathways or vacuole-membrane fusion and have shown promising potential in cancer therapy (Cui et al., 2024). For instance, Ganoderma-derived exosome-like nanovesicles have been found to enrich Fe^2+^, promote the Fenton reaction, upregulate ALOX15, inhibit GPX4 activity, and enhance lipid peroxidation, thereby disrupting the antioxidant defense system and inducing ferroptotic cell death in HCC cells (Chu et al., 2024). Similarly, exosome-like nanovesicles isolated from fresh tea leaves are efficiently internalized by 4T1 breast cancer cells, significantly increasing intracellular ROS levels, inducing mitochondrial damage, and triggering apoptosis (Sha et al., 2024). Additionally, grape-derived exosome-like nanovesicles can deliver natural polyphenols, downregulate GPX4, upregulate transferrin receptor 1 (TFR1), and enhance iron uptake, leading to excessive ROS accumulation and ferroptosis, effectively inhibiting colorectal cancer cell proliferation (Dad et al., 2021). These PDENs show great promise in inhibiting tumor cell proliferation or inducing programmed death. The therapeutic potential of these agents in HCC management necessitates comprehensive evaluation, particularly regarding their role in ferroptosis-targeted intervention strategies.

In fundamental research, *F. dibotrys* extract has demonstrated significant antitumor effects. For instance, an injectable hydrogel composed of *F. dibotrys* extract-loaded gellan gum enables localized chemotherapy through Ca^2+^ crosslinking (Xie et al., 2023). FdNVs were successfully obtained via ultracentrifugation, and NTA revealed a hydrodynamic diameter of 168.7 nm, indicating their characteristic vesicle morphology and good dispersion. These physicochemical characteristics enable FdNVs to exploit the enhanced permeability and retention phenomenon, facilitating tumor-targeted deposition and positioning them as a promising platform for nanoparticle-based therapeutic delivery owing to their sub-200 nm dimensions (Yang R. et al., 2024). Notably, Dil-labeling experiments confirmed that FdNVs were internalized by HepG2 cells, with a significant accumulation of fluorescence in the cytoplasm, suggesting endocytosis-mediated uptake. This process may involve membrane fusion facilitated by vesicular surface proteins (Liang et al., 2018). The CCK-8 assay revealed that FdNVs exert a concentration-dependent inhibitory effect on HepG2 cells. This finding was further corroborated by results from the EdU and colony formation assays. Moreover, wound healing and invasion assays demonstrated that FdNVs significantly attenuated the migratory and invasive capabilities of HepG2 cells.

Based on these findings, we co-treated HepG2 cells with FdNVs and inhibitors targeting multiple programmed cell death (PCD) pathways. The findings indicated that the ferroptosis inhibitor alone effectively reversed the reduction in cell viability caused by FdNVs, suggesting that ferroptosis is the predominant mechanism underlying FdNVs-induced cell death. A hallmark of ferroptosis is intracellular Fe^2+^ overload, which drives explosive ROS production via the Fenton reaction. Concurrently, inhibition of the cystine/glutamate antiporter (System Xc^−^) leads to impaired GSH synthesis and a significant reduction in GPX4 activity, ultimately triggering a peroxidation cascade of polyunsaturated fatty acid phospholipids (PUFA-PLs) (Fan et al., 2025). The peroxidation of PUFAs is a defining event in ferroptosis. Due to their bis-allylic structure, PUFAs are highly susceptible to oxidation, generating lipid peroxidation products such as MDA and 4-hydroxynonenal (4-HNE), which further compromise cell membrane integrity (Qiu et al., 2024). Additionally, PUFA peroxidation is regulated by key enzymes, including ACSL4 and LPCAT3. ACSL4 activates PUFAs by converting them into PUFA-CoA, which is subsequently incorporated into membrane phospholipids by LPCAT3, rendering them more prone to oxidation. These PUFA-enriched phospholipids undergo oxidation either via the Fenton reaction or enzymes such as lipoxygenases (LOX), ultimately leading to membrane rupture and cell death (Zheng et al., 2024; Fernández-Acosta et al., 2025). To assess the extent of Fe^2+^ accumulation and lipid peroxidation, we measured intracellular ROS levels, Fe^2+^ fluorescence intensity, MDA content, and GSH levels across different treatment groups. The results demonstrated that FdNVs-treated cells exhibited significantly increased ROS levels, Fe^2+^ accumulation, and MDA content, while GSH levels were markedly reduced. Furthermore, we utilized the JC-1 fluorescence probe to evaluate MMP and performed TEM to examine ultrastructural changes. JC-1 staining revealed that FdNVs induced a disruption of MMP, leading to mitochondrial dysfunction. TEM analysis showed characteristic mitochondrial shrinkage, cristae fragmentation, cristae reduction or disappearance, and outer membrane damage, further confirming FdNVs-induced mitochondrial damage. Collectively, these results indicate that FdNVs induce ferroptosis in HepG2 cells in a dose-dependent manner by promoting lipid peroxidation and mitochondrial dysfunction.

p53 is a crucial tumor suppressor that regulates cellular redox balance through multiple mechanisms during ferroptosis. One of its key functions is to transcriptionally suppress the expression of SLC7A11, a key subunit of the xCT responsible for cystine uptake into the cell for GSH synthesis (Wang et al., 2024). Activation of p53 directly downregulates SLC7A11, leading to a reduction in cystine uptake. Since cystine is a rate-limiting precursor for GSH synthesis, the inhibition of SLC7A11 significantly decreases intracellular GSH levels. GPX4, a key ferroptosis regulator, relies on GSH as a cofactor to neutralize lipid peroxides and maintain oxidative homeostasis in cells. GSH depletion leads to GPX4 inactivation, preventing the elimination of lipid peroxides. This results in the accumulation of oxidized lipid species, coupled with increased ROS levels. Meanwhile, Fe^2+^ participates in the Fenton reaction, generating abundant lipid radicals, which further disrupt the integrity of the cell membrane, leading to osmotic imbalance, cell swelling, and ultimately ferroptotic cell death (Huang et al., 2025). Furthermore, ALOX15, a key lipoxygenase enzyme, is essential for catalyzing the peroxidation of polyunsaturated fatty acid phospholipids. Elevated levels of ALOX15 directly enhance PUFA oxidation, thereby exacerbating lipid peroxidation pathways. Acting as a transcription factor,p53 can directly interact with the ALOX15 promoter, boosting its transcriptional activity and subsequently increasing ALOX15 expression at both the mRNA and protein levels (Song et al., 2024). This regulatory pathway may interact synergistically with the recently characterized p53-SAT1-ALOX15 axis (Yang H. et al., 2024). In our study, we assessed the expression levels of key ferroptosis-related mRNAs and proteins using RT-qPCR and Western blot analysis. The results demonstrated that FdNVs significantly upregulated p53 and ALOX15 expression at both the mRNA and protein levels, while concurrently downregulating SLC7A11 and GPX4. These findings confirm that FdNVs induce ferroptosis in HCC cells via the p53/xCT/GPX4 signaling pathway.

Furthermore, *in vivo* experiments using subcutaneous tumor xenografts in nude mice yielded consistent results with *in vitro* findings. FdNVs treatment significantly suppressed tumor growth, as evidenced by HE staining, which revealed large areas of necrosis in tumor tissues. Ki67 expression was markedly reduced, indicating diminished tumor cell proliferation. TUNEL assay results showed a significant increase in apoptotic cells, suggesting that apoptosis may be a secondary effect following ferroptosis induction, further validating the antitumor efficacy of FdNVs. Additionally, intratumoral ROS levels progressively increased in a dose-dependent manner. Both high-dose FdNVs and Sorafenib induced excessive ROS accumulation, indicating that FdNVs exert their antitumor effects by promoting oxidative stress. Western blot analysis further corroborated these findings, showing that FdNVs significantly upregulated p53 and ALOX15 expression, while downregulating GPX4 and xCT protein levels. These findings provide further evidence that FdNVs induce ferroptosis in HCC via the p53/xCT/GPX4 signaling axis.

## Conclusion

This study systematically elucidates the molecular mechanism by which FdNVs target the p53/xCT/GPX4 signaling axis to induce ferroptosis in HCC cells. The findings provide experimental evidence supporting the development of a natural, low-toxicity, and highly effective therapeutic strategy for HCC, introducing a novel ferroptosis-based approach for cancer treatment. In the future, by integrating the advantages of modern medicine and traditional Chinese medicine, this strategy holds the potential to offer significant clinical benefits for HCC patients and further advance the field of liver cancer treatment.

## Data Availability

The raw data supporting the conclusions of this article will be made available by the authors, without undue reservation.
