# Supplementary figures and images for: Molecular mechanisms underlying Fagopyrum dibotrys-derived nanovesicles induced ferroptosis in hepatocellular carcinoma: a dual-pathway analysis of lipid peroxidation and mitochondrial damage
Source: Front Pharmacol. 2025 Jun 26;16:1636149. doi: 10.3389/fphar.2025.1636149 (PMC12241094; doi:10.3389/fphar.2025.1636149)

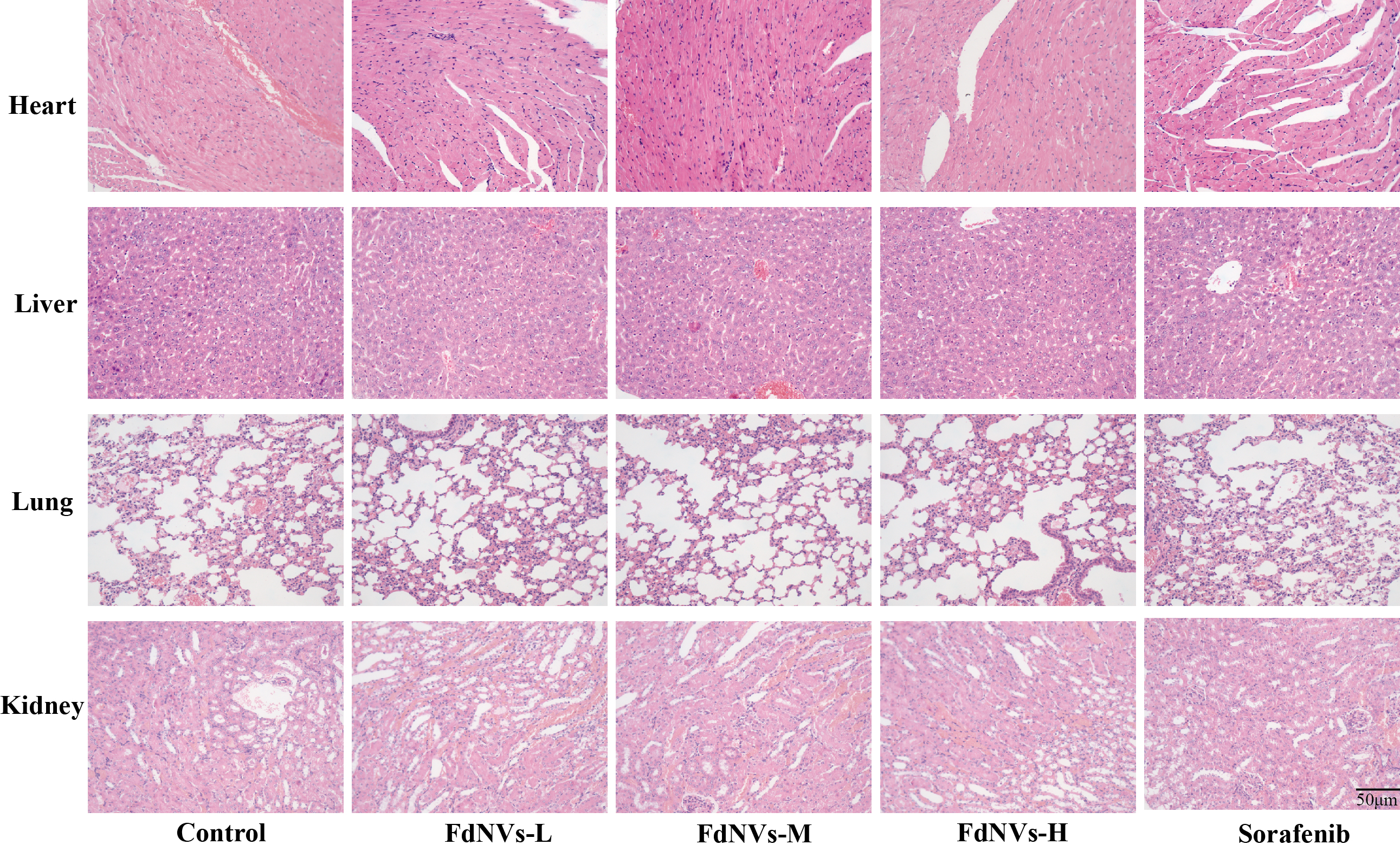

Supplement: Supplementary file 1 [file Image1.jpeg]
